# Gene expression and differentiation characteristics in mice E13.5 and E17.5 neural retinal progenitors

**Published:** 2009-12-02

**Authors:** Xuerong Sun, Ruzhang Jiang, Yuehong Zhang, Mengfei Chen, Peng Xiang, Ying Qi, Qianying Gao, Bing Huang, Jian Ge

**Affiliations:** 1 State Key Laboratory of Ophthalmology, Zhongshan Ophthalmic Center, Sun Yat-sen University, Guangzhou, China; 2 Center for Stem Cell Biology and Tissue Engineering, Sun Yat-sen University, Guangzhou, China

## Abstract

**Purpose:**

Retinal progenitor cells (RPCs) are the most valuable seed cells in replacement therapy for neural retinal diseases. The competence of RPCs changes with retinal development. Gene expression plays a fundamental role in determining the competence. To improve the selection of the right-timing RPCs for replacement therapy, we compared the gene expression between embryonic day (E) 13.5 and E17.5 RPCs and further explored their gene expression and differentiation capacity in vitro.

**Methods:**

Timed-pregnant E13.5 and E17.5 RPCs were freshly harvested and cultured in proliferation conditions for 4 days and then in differentiation conditions for 8 days. At different time points, the expression of key genes involved in retinal development was investigated by quantitative reverse transcription-PCR or immunofluorescence.

**Results:**

The expression of 14 key genes involved in retinal development was investigated in freshly harvested E13.5 and E17.5 RPCs. The freshly harvested E13.5 RPCs showed a high expression of retinal ganglion cell (RGC)-related genes, including *Math5, Brn3b, Islet1*, and *Nfl*, while the freshly harvested E17.5 RPCs displayed a high expression for *Nrl, GFAP*, and *Thy1*, the key genes involved in rod photoreceptor development, glial cell development, and synaptogenesis, respectively. During proliferation culture in vitro, the gene expression changed dramatically in both RPCs. After the 4 days of proliferation culture, the expression levels of most genes (11 of the 14 genes) in E13.5 RPCs came close to those in the freshly harvested E17.5 RPCs. Differentiation of RPCs in vitro was verified by the significant decrease in *Nestin* expression and BruU incorporation efficiency. After the 8 days of differentiation in vitro, the expression level of RGC-related genes (*Math5, Brn3b*, and *Islet1*) was still significantly higher in E13.5 RPCs than in E17.5 RPCs. In contrast, the expression level of *Nrl* and *GFAP* was significantly higher in E17.5 RPCs than in E13.5 RPCs. In morphology, the differentiated E13.5 RPCs displayed more robust process outgrowth than did the differentiated E17.5 RPCs. Immunofluorescence showed that, after the 8 days of differentiation, E13.5 RPCs contained more *Brn3b*- and *Map2*-positive cells, while E17.5 RPCs contained more *GFAP*-, *GS*-, and *Rhodopsin*-positive cells.

**Conclusions:**

The results implied that E13.5 RPCs might be a better choice for RGC replacement therapy, while E17.5 RPCs might be better for photoreceptor replacement therapy. The duration of in vitro culture should be timed, since the expression of key genes kept changing in the proliferating RPCs.

## Introduction

The degeneration and loss of neural retinal cells, especially retinal ganglion cells (RGCs) and photoreceptor cells, is a common result of many retina-related diseases, including glaucoma, age-related macular degeneration, and diabetic retinopathy. Millions of people in the world suffer blindness as a result of retinal cell loss. At present, no treatment can reverse the degeneration of neural retinal cells. In recent laboratory research, replacement therapy of lost retinal cells by stem cell transplantation has proven to be promising. Among various stem cells, retinal progenitor cells (RPCs) are among the best seed cells for attaining functional integration after transplantation [1-3].

In the past thirty years, great work has been done in exploring the in vivo behavior of RPCs. The competence of RPCs changes in an evolutionarily conserved temporal sequence during retinal development [4]. In early embryonic stages, RPCs mainly give birth to retinal ganglion cells (RGCs), cone photoreceptors, horizontal cells, and amacrine cells, while in late embryonic and neonatal stages, RPCs mainly produce rod photoreceptors, bipolar cells, and Muller cells [5-7]. In the determination of RPC competence, intracellular gene expression, especially that of transcription factors, plays a fundamental role [4,8-10]. Since competence is one of the most valuable biologic parameters in stem cell applications, the changing competence of RPCs reminds us of the importance of selecting right-timing RPCs [1,11,12].

RPCs mainly reside in either the embryonic retina or the adult ciliary margin zone [13-15]. Harvesting sufficient RPCs is necessary for in vivo transplantation, but is often difficult. Therefore, the proliferation culture of RPCs in vitro is necessary in many situations. However, it is still unclear whether and how the competence changes in proliferation-cultured RPCs.

Mice at embryonic day E13.5 and E17.5 represent two stages in retinal development. E13.5 is in the early developmental stages, during which the generation of early retinal cells, especially RGCs, is vigorous. E17.5 is in the late stages, during which the birth of rod photoreceptors, bipolar cells, and glial cells is speeding up [5,6].

To improve the selection of the right-timing RPCs for replacement therapy, we compared the gene expression and the differentiation capacity of E13.5 and E17.5 RPCs. Both freshly-harvested and in vitro-cultured RPCs were investigated to inspect whether the competence of RPCs changed not only with development, but also with the duration of in vitro culture.

## Methods

### Animal preparation and cell culture

Pregnant mice (SPF grade) were obtained from the Center of Experimental Animals Sun Yat-sen University, China. Thirty-two pregnant mice were used in the study. All experimental procedures were conducted in conformity with the institutional guidelines for the care and use of laboratory animals in Zhongshan Ophthalmic Center, Sun Yat-sen University, and complied with the ARVO Statement for the Use of Animals in Ophthalmic and Visual Research.

At E13.5 or E17.5, the pregnant mice were euthanized by cervical vertebra dislocation. The harvesting and culture of retinal progenitor cells was done according to Wang [5] and Yao [16] with minimal modification. Briefly, the anterior eye segment was removed from each of the embryonic mice under a dissecting microscope (MZ6, Leica, Bensheim, Germany), and the neural retinal tissue was separated from the pigmented epithelial layer. The neural retinal tissue was then digested using TrypLE Express (Gibco, Grand Island, NY) for 10 min at 37 °C and then stopped with Dulbecco’s Modified Eagle Medium (DMEM)/F12. After centrifugation, cell pellets were resuspended with neural growth medium and dissociated by pipetting repeatedly through a fire-polished pipette. The cell suspension was then seeded onto an uncoated 6-well plate at a density of 2×10^5^ cells/ml and cultured in a 37 °C incubator containing 5% CO_2_. The neural growth medium for RPC proliferation was serum free and composed of DMEM/F12, 1% GlutaMax (Gibco), 2% B27 supplement (Gibco), 1% N2 supplement (Gibco), 20 ng/ml bFGF (R&D Systems, Minneapolis, MN), and 20 ng/ml EGF (PeproTech, Rocky Hill, NJ). EGF and bFGF were added to the medium every 3 days.

In this study, two kinds of culture methods were adopted for RPCs. For proliferation culture, RPCs were seeded onto an uncoated plate and cultured with neural growth medium. The differentiation culture was following established procedure [17,18], with minimal modification. Briefly, RPCs were dissociated by trypsinization and pipetting, and then 1×10^4^ cells were plated onto Poly-D-lysine (PDL)-coated coverslips and cultured in a modified neural growth medium with bFGF and EGF removed and supplemented with 2% fetal bovine serum (FBS).

### Real-time quantitative reverse transcription-PCR

Total RNA was collected from freshly harvested or cultured RPCs. The procedure for extracting total RNA was performed according to the instructions in the RNeasy Mini Kit (Qiagen, Hilden, Germany). DNase І (Sigma, St. Louis, MO) treatment was then used to reduce the likelihood of genomic DNA contamination. cDNA was synthesized using the PrimeScript RT reagent Kit (TaKaRa, Tokyo, Japan). For quantitative PCR, SYBR Premix Ex Taq (Takara) was used. In each PCR reaction system (20 μl), the mixture contained cDNA template (approximately 50 ng), SYBR Premix Ex Taq, forward and reverse primers (0.2 μm), and ROX Reference Dye. The reaction was conducted in an ABI Prism 7000 Real-Time PCR System with the following program: 1×95 °C, 10 s; 1×95 °C, 5 s; 40×60 °C, 31 s. After amplification, melting curve analysis was performed to confirm the specificity of PCR products. Table 1 shows the primer pairs used in the experiment. The expression level of each gene was calculated by normalizing it with the *GAPDH* gene, which served as an internal reference, as reported in [19]. The mRNA level of each gene (x) relative to *GAPDH* was calculated as follows [20]: mRNA (x/*GAPDH*)=2^Ct(^*^GAPDH^*^)-Ct(x)^. The relative mRNA level between different samples (a and b, for example) was calculated as follows: mRNA (a/b)=2^ΔCt(b)-ΔCt(a)^; ΔCt(a,b)=Ct_(x)_-Ct_(GAPDH)_. Data was analyzed using STATA 10.0.

**Table 1:** Sequence of primer pairs used in real-time quantitative RT–PCR

**Gene**	**Primer sequence (5′-3′)**	**Product (bp)**	**GenBank accession number**	**Gene function**
*Pax6*	F: CAGCTTCACCATGGCAAACAAC	116	NM_013627	RPCs development [4]
	R: AGGTATCATAACTCCGCCCATTCA			
*Rx1*	F: CTTACCAACTGCACGAGCTGGA	137	NM_013833	RPCs development [4]
	R: CTTAGCCCGTCGGTTCTGGA			
*Nestin*	F: GGGCCAGCACTCTTAGCTTTGATA	105	NM_016701	Neural progenitors marker
	R: TGAGCCTTCAGGGTGATCCAG			
*Nfl*	F: TCAATGTCAAGATGGCCTTGGA	99	NM_010910	Immature neuron marker
	R: TTATGCTACCCACGCTGGTGAA			
*Math5*	F: GAAGCTGTCCAAGTACGAGACACTG	100	NM_016864	RGCs development [33,34]
	R: GTGAGCGCGATGATGTAGCTG			
*Brn3b*	F: CGATGCGGAGAGCTTGTCTTC	132	NM_138944	RGCs development [33,35]
	R: GATGGTGGTGGTGGCTCTTACTCT			
*Islet1*	F: CAGACCACGATGTGGTGGAGA	109	NM_021459	RGCs development [33]
	R: TGCCTAGCCGAGATGGGTTC			
*GFAP*	F: ACCAGCTTACGGCCAACAGTG	139	NM_010277	Glial cells development
	R: TGTCTATACGCAGCCAGGTTGTTC			
*Nrl*	F: ACGACCTGGGCAGTAGTCTCAA	109	NM_008736	Rod photoreceptors development [36]
	R: GTGTCGGAAGTCATCCAGTTCAA			
*Thy1*	F: CACCAAGGATGAGGGCGACTA	118	NM_009382	Synaptogenesis [37,38]
	R: GCTTATGCCGCCACACTTGA			
*Foxn4*	F: CGAGCGAGCACTTTGGTGAC	94	NM_148935	Amacrine development [39]
	R: AGGAGCAGATGTGAGCCATGATAA			
*Delta1*	F: AGGGTGTGATGACCAACATGGA	96	NM_007865	Notch pathway
	R: TATCGGATGCACTCATCGCAGTA			
*Notch1*	F: CTCCAACTGTGACACCAACC	108	NM_008714	Notch pathway
	R: GCACCCAGATCACACTCATC			
*Hes1*	F: CCAATTTGCCTTTCTCATCC	112	NM_008235	Notch pathway
	R: GGAAGGTGACACTGCGTTAG			
*GAPDH*	F: CCTGCGACTTCAACAGCAACTC	119	NM_008084	Housekeeping gene
	R: GTTGCTGTAGCCGTATTCATTGTCA			

### Bromodeoxyuridine labeling

To label the proliferation- and differentiation-cultured RPCs, 1 µM BrdU (B-9285; Sigma) was added to the medium to incubate for 48 h. Then the BrdU was removed by rinsing twice with PBS solution. The proliferation-cultured RPCs were replated onto PDL-coated plates to allow attachment for another 4–6 h. The labeled RPCs were fixed with 4% paraformaldehyde before immunofluorescence detection.

### Immunofluorescence

RPCs were collected and fixed in 4% paraformaldehyde for 15 min at room temperature. After blocking with 4% BSA in PBS containing 0.2%–0.5% Triton X-100 for 1 h, RPCs were incubated with the primary antibody, which included mouse anti-Nestin (1:100; MAB353; Millipore, Temecula, CA), mouse anti-BrdU (1:50; MAB3510; Millipore), goat anti-Brn3b (1:200; sc-31987; Santa Cruz Biotechnology, Santa Cruz, CA), mouse anti-GFAP (1:300; MAB353; Millipore), mouse anti-Map2 (1:50; Boster, Wuhan, China), rabbit anti-rhodopsin (1: 200; MAB5356; Chemicon), and rabbit anti-glutamine synthetase (1: 50; ab16802; Abcam, Cambridge, UK). After incubating with primary antibodies at 4 °C overnight, RPCs were rinsed 5 min in PBS for 4 times. Then they were incubated with the secondary antibodies, including rhodamine-conjugated anti-goat IgG (1:200; sc-2094; Santa Cruz), Cy3-conjugated anti-mouse IgG (1:100; C2181; Sigma), Alexa Fluor 488-conjugated anti-mouse IgG (1:800; A-21202; Molecular Probes) and Cy3-conjugated anti-rabbit IgG (1:50; BA1032; Boster), for about 1 h at room temperature. Hoechst 33342 (Invitrogen, Carlsbad, CA) was used for counterstaining. A negative control was performed by replacing the primary antibody with goat serum (16210–064; Gibco), mouse serum (10410; Invitrogen), or rabbit serum (16120–099; Gibco). Fluorescence was excited and detected using a confocal microscope (LSM 510 MEH, Carl Zeiss, Jena, Germany) or fluorescence microscope (Axioplan 2, Zeiss). Each experiment was repeated twice. Image-Pro Plus 6.0 software was used to analyze the results.

## Results

### Gene expression in E13.5 retinal progenitor cells relative to E17.5 retinal progenitor cells

**Figure 1:**
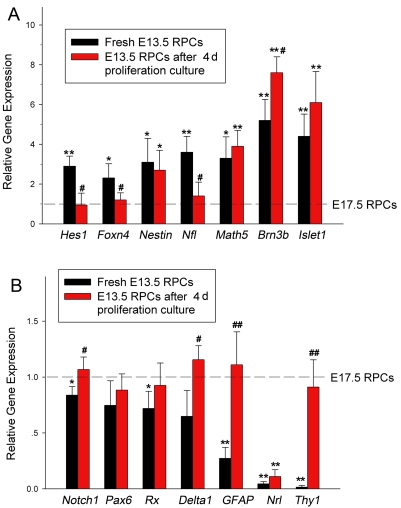
Gene expression in freshly harvested or proliferation-cultured E13.5 retinal progenitor cells (RPCs) relative to fresh E17.5 RPCs. Expression of each gene in freshly harvested E17.5 RPCs was defined as 1. The columns represent the relative expression level in E13.5 RPCs, which were either freshly harvested (black column) or proliferation-cultured for 4 days (red column). Panel **A** shows the genes whose initial expression was high in fresh E13.5 RPCs, but mostly decreased after 4 days culture. Panel **B** represents those genes whose initial expression was low in fresh E13.5 RPCs, but increased after 4 days culture. Note that the expression change of *Math5, Brn3b*, and *Islet1* in E13.5 RPCs was exceptional. The symbols * and ** represent p<0.05 and p<0.01, respectively, versus fresh E17.5 RPCs. The symbols # and ## represent p<0.05 or p<0.01, respectively, versus fresh E13.5 RPCs. Data was mean±standard deviation (n=4). The significance was estimated by one-way ANOVA.

To learn about the differential competence between freshly harvested E13.5 and E17.5 RPCs in molecular level, the expression of 14 key genes involved in retinal development was investigated by quantitative reverse transcription-PCR (RT–PCR). The expression level of each gene in fresh E17.5 RPCs was defined as 1. The results showed that the expression of *Nestin* (neural progenitor marker), *Hes1* (Notch1 effector), and the retinal ganglion cell (RGC)-related genes, including *Math5, Brn3b, Islet1*, and *Nfl*, were significant higher in E13.5 RPCs than in E17.5 RPCs (n=4, Figure 1A, black column). In contrast, *Thy1, Nrl*, and *GFAP*, which are involved in synaptogenesis, the development of the rod photoreceptors, and the development of the glial cells, respectively, showed higher expression in E17.5 RPCs (n=4, Figure 1B, black column). The gene expression patterns in E13.5 and E17.5 RPCs were generally consistent with the birth of stage-specific retinal cells in vivo [5,6].

Proliferation-culture of RPCs in vitro is often necessary to obtain enough seed cells. The developmental interval between E13.5 and E17.5 is 4 days. To explore the change of gene expression in vitro, the freshly-harvested E13.5 RPCs were proliferation-cultured in vitro for 4 days before the gene expression was detected by quantitative RT–PCR. The results showed that the expression level of most genes changed. In the 14 genes detected, the expression levels of 11 genes in E13.5 RPCs were closer to those in the freshly harvested E17.5 RPCs, including *GFAP, Nrl, Nfl, Pax6*, and *Rx* (Figure 1). Though not all the changes were statistically significant, which may have been due to the small sample size, the tendency to change toward E17.5 RPCs was obviously. The tendency to change in gene expression implied that, when proliferation-cultured in vitro, E13.5 RPCs could continue their intrinsic development to some extent, though not as completely as in vivo.

Interestingly, the expression of several RGC-related genes, *Math5, Brn3b*, and *Islet1*, increased in E13.5 RPCs after 4 days of proliferation (Figure 1A), which was opposite the developmental direction of 17.5 RPCs. The unexpected results might partially have resulted from the purification effect of the suspension culture, which will be discussed in the following section.

### Proliferation- and differentiation-culture of E13.5 and E17.5 RPCs in vitro

To explore the gene expression and differentiation capacity of E13.5 and E17.5 RPCs in vitro, we harvested the freshly isolated E13.5 and E17.5 RPCs and cultured them in proliferation and differentiation conditions.

**Figure 2:**
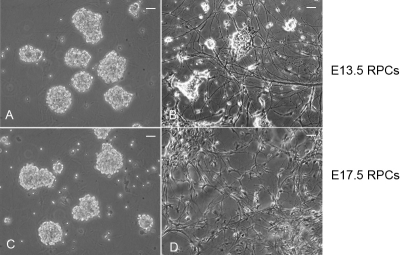
E13.5 and E17.5 RPCs cultured in proliferation and differentiation conditions. Both RPCs adopted suspension growth and formed neurospheres in proliferation culture (**A**, **C**). After starting the differentiation culture, E13.5 and E17.5 RPCs became adherent growth and initiated differentiation by displaying stretched-out processes (**B**, **D**). Note that the processes in E13.5 RPCs (**B**) were long and thick, while in E17.5 RPCs (**D**) they were mainly short and thin. Bars were 50 μm.

**Figure 3:**
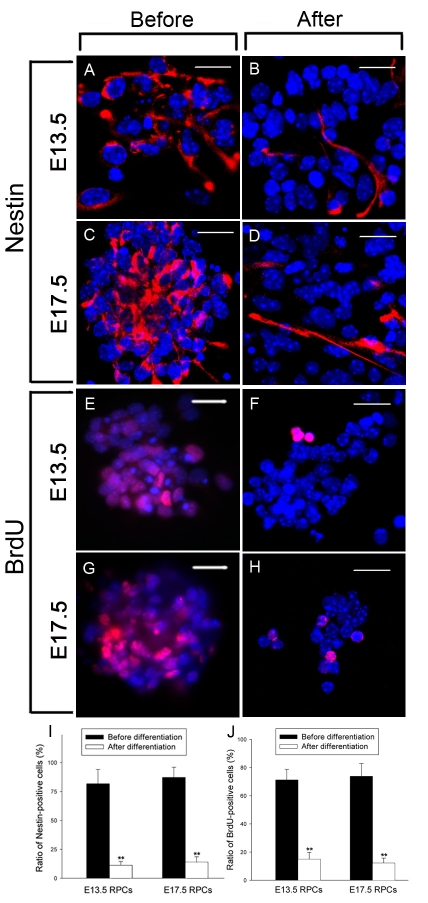
*Nestin* expression and BrdU incorporation in RPCs before and after differentiation. The 4 day proliferation-cultured E13.5 and E17.5 RPCs were of differentiation-cultured. Before and after 8 day of differentiation, *Nestin* expression and BrdU incorporation were detected. Note that before differentiation, the expression of Nestin in both RPCs was vigorous, but significantly decreased after 8 days of differentiation (**A–D**, **I**). Similarly, the incorporation ratio of BrdU in both RPCs decreased dramatically after differentiation (**E–H**, **J**). Bars were 20 μm. Data in **I** and **J** is mean±standard deviation (n=3). The significance was determined by the Student *t*-test. The symbol ** represents p<0.01 versus before differentiation.

**Figure 4:**
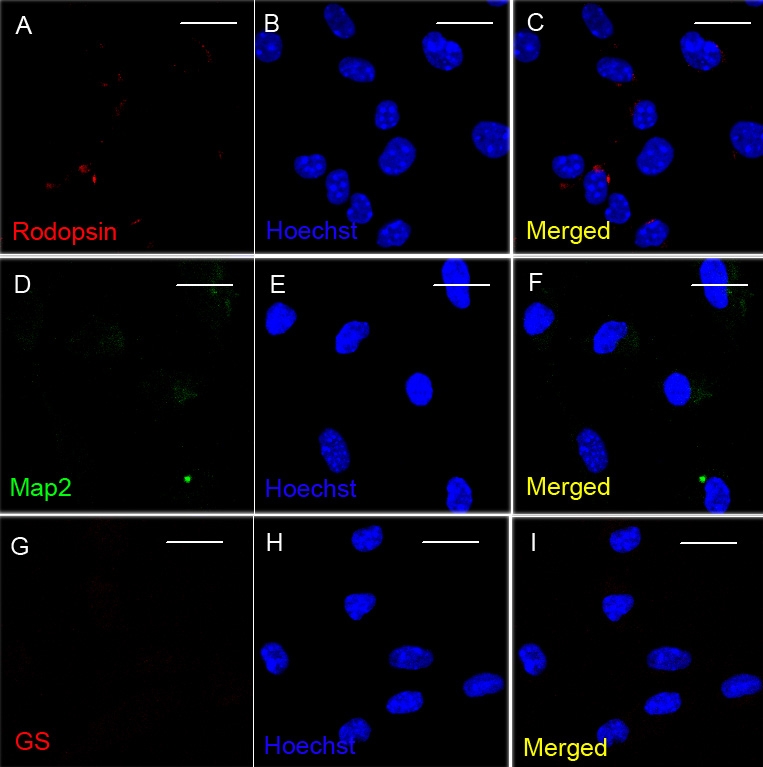
Detection of the mature retinal markers in the proliferation-cultured E13.5 RPCs. After 4 day proliferation culture in vitro, E13.5 RPCs showed no obvious expression of the mature retinal markers *Rhodopsin* (**A-C**), *Map2* (**D-F**), or *GS* (**G-I**). Bars were 20 μm.

In the proliferation culture, both E13.5 and E17.5 RPCs began suspension growth and formed neurospheres (Figure 2). Some researchers have indicated that the RPCs in these developmental stages are mostly immature progenitor cells [6,17,21]. To confirm that the RPCs in our study were still in an undifferentiated state after 4 days of proliferation culture, expression of Nestin, a neural progenitor marker, was investigated. The results showed that most cells in E13.5 and E17.5 RPCs expressed Nestin (82% and 87%, respectively; Figure 3). Similarly, about 71% and 74% cells in E13.5 and E17.5 RPCs, respectively, incorporated BrdU after 48 h exposure (Figure 3). Furthermore, no evident expression of mature retinal/neural marker could be detected in the 4 days proliferation-cultured RPCs (Figure 4). These results suggested that both RPCs were still in an undifferentiated state, and that they continued to actively renew after 4 days of proliferation culture.

To induce RPC differentiation, the suspended neurospheres were dissociated by trypsinization and then seeded onto a PDL-coated coverslip to allow attachment. Differentiation of both RPCs was confirmed by the significant downregulation of Nestin expression and the BrdU incorporation ratio (Figure 3). The decrease of Nestin during RPC differentiation was similar to that previously reported [22]. Morphologically, after being differentiation-cultured for about 8 days, E13.5 RPC stretched out long and thick processes, while E17.5 RPCs mainly displayed short and thin processes (Figure 2).

### Gene expression in E13.5 and E17.5 RPCs under proliferation and differentiation conditions

**Figure 5:**
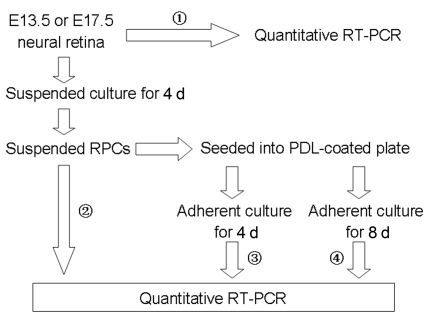
Diagram of gene expression detection in E13.5 and E17.5 RPCs under conditions of proliferation and differentiation culture. Total RNA was collected from four sources (circled numbers). Gene expression was detected by quantitative RT–PCR. To induce RPC differentiation, 2% fetal bovine serum was added to the growth medium in the adherent culture.

The differential gene expression between freshly harvested E13.5 and E17.5 RPCs verified their different competences [5,6]. To explore their specific differentiation capacity in vitro, the freshly harvested E13.5 and E17.5 RPCs were first proliferation-cultured for 4 days to allow some expansion, and then differentiation-cultured for 4 days or 8 days. The expression of some key genes involved in retinal development was explored at these points in time. The diagram of the experimental procedure is shown in Figure 5.

**Figure 6:**
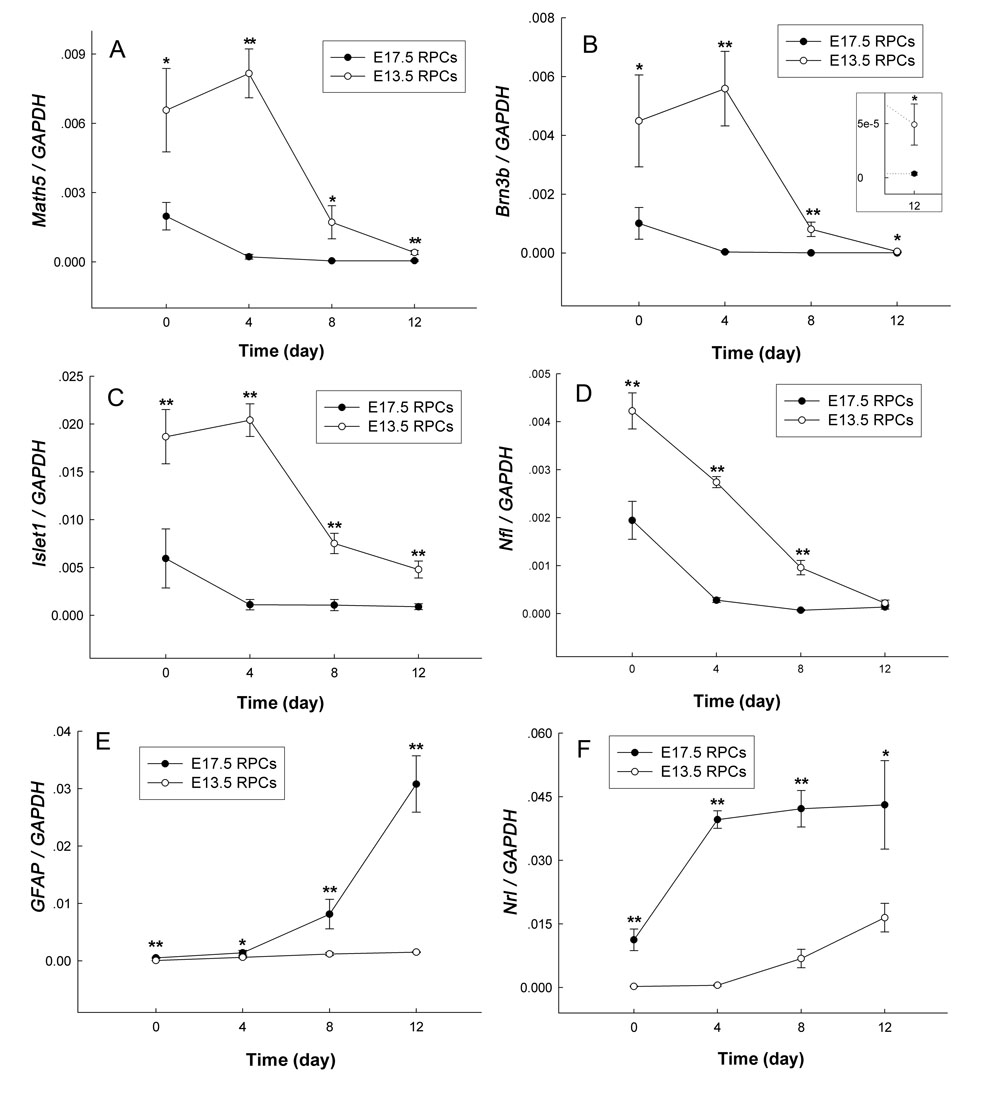
Gene expression in E13.5 and E17.5 RPCs under conditions of proliferation and differentiation culture. At 0 d: freshly harvested RPCs; 0–4 d: proliferation culture; 4–12 d: differentiation culture. (The detailed experimental procedure is shown in Figure 5.) *GAPDH*, a housekeeping gene, served as an internal control. The insert in panel **B** was the local magnification at 12 days. Note the deviation bars at some points were almost indiscernible due to the large scale of the y-axis. The values were mean±standard deviation from three experiments. The symbols * and ** represent p<0.05 and p<0.01, respectively, versus E17.5 RPCs (**A-D**) or E13.5 RPCs (**E-F**). The significance was determined by the Student *t*-test.

**Figure 7:**
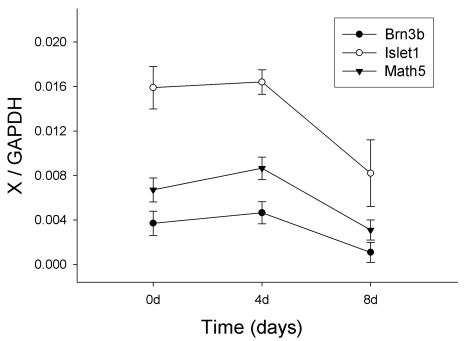
RGCs-related genes expression in E13.5 RPCs during 8 day proliferation culture The “X” letter in the y-axis represents *Brn3b, Math5, or Islet1*. *GAPDH* served as an internal control. Note that the expression of *Brn3b, Math5*, and *Islet1* weakly increased after 4 day culturing, followed by a dramatic decrease after 8 day. Data was mean±standard deviation (n=3).

Figure 6 shows the dynamic change in gene expression. *Math5, Brn3b*, and *Islet1* are key genes involved in RGCs development. Expression of these genes in E13.5 RPCs increased after 4 days of proliferation culture and then dramatically decreased during the 8 days of differentiation culture (Figure 6A-C). The increase of *Math5, Brn3b*, and *Islet1* in E13.5 RPCs was exciting because it implied enrichment of retinal ganglion progenitors. Nevertheless, prolonging the proliferation culture up to 8 days showed that the increased expression of these genes could not be maintained (Figure 7). On the other hand, the expression of these RGC-related genes in E17.5 RPCs kept decreasing both in proliferation and differentiation cultures (Figure 6A-C). Though the expression of *Math5, Brn3b*, and *Islet1* evidently decreased in both RPCs during differentiation culture, the expression level of these genes was still significantly higher in E13.5 RPCs than in E17.5 RPCs after 8 days of differentiation (Figure 6A-C).

*Nfl* is another gene involved in RGCs development. The expression of Nfl kept decreasing in both RPCs, whether during proliferation or differentiation culture (Figure 6D). Unlike *Math5, Brn3b*, or *Islet1*, a transient increase of *Nfl* in proliferative E13.5 RPCs was absent. The manner of differential expression between Nfl and each of *Math5, Brn3b*, or *Islet1*, suggests that they might be expressed in different cell subgroups or in different developmental stages.

*GFAP* and *Nrl* belong to glial and rod photoreceptor markers, respectively. Expression of these genes in both RPCs increased continuously during the proliferation and differentiation cultures (Figure 6E-F). During differentiation culture, the increasing velocity of *GFAP* in E17.5 RPCs was obviously faster than in E13.5 RPCs (Figure 6E), suggesting that E17.5 RPCs were more prone to differentiate toward glial cells than E13.5 RPCs. After 8 days of differentiation, expression of *GFAP* and *Nrl* was significantly higher in E17.5 RPCs than in E13.5 RPCs (Figure 6E-F).

### Protein expression in differentiated E13.5 and E17.5 RPCs

To further explore the differentiation capacity of E13.5 and E17.5 RPCs in protein levels, both RPCs were induced to differentiate following the methods in Figure 5. After 8 days, the expression of specific retinal markers was detected by immunofluorescence.

**Figure 8:**
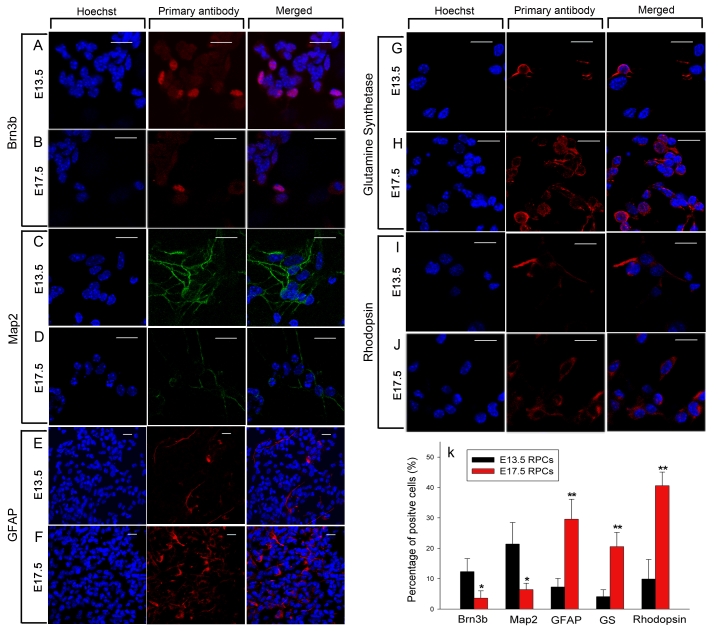
Immunofluorescence detection in differentiated E13.5 and E17.5 RPCs. After 8 days of differentiation, the expression of *Brn3b* (panel **A**, **B**), *Map2* (panel **C**, **D**), *GFAP* (panel **E**, **F**), glutamine synthetase (panel **G**, **H**), and Rhodopsin (panel **I**, **J**) in E13.5 and E17.5 RPCs was investigated. **K** is the statistical ratio of positive cells in both RPCs. Note that the expression ratios of *Brn3b* and *MAP2* in E13.5 RPCs were significantly higher than in E17.5 RPCs. In contrast, E17.5 RPCs expressed *GFAP*, glutamine synthetase (*GS*), and rhodopsin in higher percentages. The values were mean±standard deviation from three experiments. The symbols * and ** represent p<0.05 and p<0.01, respectively, versus E13.5 RPCs. Bars were 20 μm. The significance was evaluated by the Student *t*-test.

The results showed that the expression of the RGCs marker Brn3b and the neuronal marker Map2 in E13.5 RPCs was significantly higher than that in E17.5 RPCs (12.3% versus 3.6%, and 21.4% versus 6.4%, respectively; Figure 8A-D,K). In contrast, the rod photoreceptor marker rhodopsin, the glial marker GFAP, and the Muller cell marker glutamine synthetase (GS) showed significantly higher expression in E17.5 RPCs (Figure 8E-J,K). Morphologically, the GFAP- and glutamine synthetase-positive cells had a typical glial appearance.

## Discussion

### Gene expression and the competence of RPCs

Some researchers have quantitatively investigated the gene expression in RPCs at different developmental stages [17,23]. However, in most of this research, gene expression was investigated by high-throughput gene arrays, which usually need to be confirmed by quantitative RT–PCR or in situ hybridizations [17,23]. In the current study, we investigated the relative gene expression in RPCs by real-time quantitative RT–PCR, which was more sensitive than normal gene arrays.

E13.5 and E17.5 RPCs have different competences in vivo, and give rise to different retinal cell types [5,6]. The birth of neural retinal cells results from the interaction of the retinal microenvironment and the intracellular cues of RPCs [4]. Our study of freshly harvested RPCs showed that E13.5 RPCs expressed RGC-related genes in high abundance. In contrast, the genes involved in synaptogenesis (*Thy1*) and late-stage retinal cell development, such as *Nrl* and *GFAP*, showed higher expression in E17.5 RPCs. These results were consistent with the reported competence of E13.5 and E17.5 RPCs in vivo [5,6] and further verified the fundamental role of gene expression in RPC competence.

### E13.5 and E17.5 retinal progenitor cells play different roles in retinal replacement therapy

 Many reports have held cell replacement therapy for retina-related diseases to be promising. Among various seed cells, RPCs have proved to be effective, and a better choice than others [1,2,24]. Among the heterogeneous RPCs transplanted into rod-defective retinas, Maclaren [1] found that the effective subgroup was exclusively comprised of post-mitotic rod precursors, which could integrate into host retinas and reestablish visual function. The effective cell subgroup was embodied by expressing the post-mitotic rod marker *Nrl* [1].

Our current research indicated that E13.5 RPCs had a high expression of the post-mitotic RGCs marker genes [25-27], such as *Math5, Brn3b*, and *Islet1*. This suggested that E13.5 RPCs contained more post-mitotic retinal ganglion precursors and could be a better choice for replacing degenerated RGCs, as compared to E17.5 RPCs. In contrast, E17.5 RPCs showed a high expression of the post-mitotic rod marker *Nrl,* and therefore could be a better choice for replacement therapy in the case of rod photoreceptor degeneration. The results of in vitro differentiation also supported this inference. After 8 days of differentiation, E13.5 RPCs contained more *Brn3b*- and *Map2*-positive cells, while E17.5 RPCs contained more rhodopsin-positive cells. Further investigation using an animal model would be necessary to confirm the in vivo therapeutic effect.

Expansion of RPCs in vitro is usually necessary to attain sufficient seed cells. There have been many reports that in vitro cultured RPCs could integrate into host retinas after transplantation [28-30]. However, little attention has been paid to the influence of in vitro culture on replacement therapy. Here, we can offer a preliminary conclusion based on the changes in gene expression. After 4 days of proliferation culture, expression of post-mitotic RGCs marker, including *Math5, Brn3b*, and *Islet1*, increased in E13.5 RPCs. Similarly, expression of the post-mitotic rod marker *Nrl* in E17.5 RPCs also increased after 4 days of proliferation. Therefore, the proliferation culture of E13.5 and E17.5 RPCs in vitro for 4 days may enhance their respective efficiency in replacement therapy. However, the effect of long-term proliferation culture on replacement therapy may be different. There have been reports of long-term culture resulting in reprogramming of RPCs to a more primordial state, which somewhat affected the replacement therapy [31].

### Development continued in retinal progenitor cells when proliferation-cultured in vitro

Some research shows that the proliferation-cultured RPCs could maintain their proliferation properties or differentiation capacity for a fairly long time [31,32]; however, this research is preliminary. The quantitative detection of gene expression, which is an important indicator of cell competence, was lacking.

Our results showed that gene expression changed rapidly and dramatically when E13.5 or E17.5 RPCs were proliferation-cultured in vitro. Since gene expression plays a key role in RPCs competence, we infer that the competence of RPCs should also change over time. Therefore, culture time should be an important factor to be considered before RPCs are transplanted.

The changing direction of gene expression in proliferation-cultured RPCs generally followed that of retinal development. After 4 days of expansion in vitro, most gene expression in E13.5 RPCs came close to that of fresh E17.5 RPCs. Similarly, in the proliferated E17.5 RPCs, expression of *GFAP* and *Nrl* increased, and expression of *Math5, Brn3b*, and *Islet1* decreased, after 4 days of culture. This was consistent with the developmental tendency in late-embryonic retinas.

The tendency to change in gene expression suggested that proliferation-cultured E13.5 and E17.5 RPCs could continue their development to some degree. Exceptionally, the expression of Math5, Brn3b, and Islet1 in E13.5 RPCs increased after 4 days of proliferation culture, which was opposite the developmental direction toward E17.5 RPCs. Several reasons may account for this result. The first was selective purification of E13.5 RPCs during suspension culture. RPCs derived from immature retinas were heterogeneous due to the retinal structure and the manual operation in harvesting RPCs. During the suspended-proliferation culture, some adherent cells showing epithelial-like or spindle shapes were observed, and they were excluded from the ensuing experiments. Therefore, suspension culture may be a purification procedure for RPCs. The second reason may be due to a limited amplification of retinal ganglion progenitors, since the generation of RGCs peaks from E13.5 to E15.5 [6,27]. Furthermore, maturation of RGCs during 4 days of proliferation culture could also result in the upregulation of RGC-related genes.

In theory, among the various seed cells, there should exist a cell or subgroup of cells that could most efficiently integrate into the recipient and rebuild biologic function. Finding the best seed cells and then thoroughly exploring their biologic characteristics, especially their specific gene expression, would make it possible to produce massive quantities of such cells by molecular biologic technologies. This study has shed some light on the gene expression and differentiation characteristics in mouse E13.5 and E17.5 RPCs, and has provided some clues to selecting right-timing RPCs. Further confirmation of these right-timing RPCs in an animal model will be necessary. Investigating the right-timing RPCs might be the right way to find the best seed cells for retina replacement therapy.

## References

[1] MacLaren RE Pearson RA MacNeil A Douglas RH Salt TE Akimoto M Swaroop A Sowden JC Ali RR Retinal repair by transplantation of photoreceptor precursors. Nature 2006 444 203 7 17093405 10.1038/nature05161

[2] Seiler MJ Thomas BB Chen Z Wu R Sadda SR Aramant RB Retinal transplants restore visual responses: trans-synaptic tracing from visually responsive sites labels transplant neurons. Eur J Neurosci 2008 28 208 20 18662343 10.1111/j.1460-9568.2008.06279.x

[3] Lamba DA Gust J Reh TA Transplantation of human embryonic stem cell-derived photoreceptors restores some visual function in Crx-deficient mice. Cell Stem Cell 2009 4 73 9 19128794 10.1016/j.stem.2008.10.015 PMC2713676

[4] Zaghloul NA Yan B Moody SA Step-wise specification of retinal stem cells during normal embryogenesis. Biol Cell 2005 97 321 37 15836431 10.1042/BC20040521

[5] Wang SW Mu X Bowers WJ Klein WH Retinal ganglion cell differentiation in cultured mouse retinal explants. Methods 2002 28 448 56 12507463 10.1016/s1046-2023(02)00264-5

[6] Young RW Cell differentiation in the retina of the mouse. Anat Rec 1985 212 199 205 3842042 10.1002/ar.1092120215

[7] Harada T Harada C Parada LF Molecular regulation of visual system development: more than meets the eye. Genes Dev 2007 21 367 78 17322396 10.1101/gad.1504307

[8] Ohsawa R Kageyama R Regulation of retinal cell fate specification by multiple transcription factors. Brain Res 2008 1192 90 8 17488643 10.1016/j.brainres.2007.04.014

[9] Locker M Borday C Perron M Stemness or not stemness? Current status and perspectives of adult retinal stem cells. Curr Stem Cell Res Ther 2009 4 118 30 19442196 10.2174/157488809788167382

[10] Kumar JP The molecular circuitry governing retinal determination. Biochim Biophys Acta 2009 1789 306 14 19013263 10.1016/j.bbagrm.2008.10.001 PMC2700058

[11] Reh TA Neurobiology: right timing for retina repair. Nature 2006 444 156 7 17093406 10.1038/444156a

[12] Bennett J Retinal progenitor cells–timing is everything. N Engl J Med 2007 356 1577 9 17429090 10.1056/NEJMcibr070209

[13] Tropepe V Coles BL Chiasson BJ Horsford DJ Elia AJ McInnes RR van der Kooy D Retinal stem cells in the adult mammalian eye. Science 2000 287 2032 6 10720333 10.1126/science.287.5460.2032

[14] Gu P Harwood LJ Zhang X Wylie M Curry WJ Cogliati T Isolation of retinal progenitor and stem cells from the porcine eye. Mol Vis 2007 13 1045 57 17653049 PMC2776542

[15] Djojosubroto MW Arsenijevic Y Retinal stem cells: promising candidates for retina transplantation. Cell Tissue Res 2008 331 347 57 17912553 10.1007/s00441-007-0501-8

[16] Yao J Sun X Wang Y Wang L Muller glia induce retinal progenitor cells to differentiate into retinal ganglion cells. Neuroreport 2006 17 1263 7 16951566 10.1097/01.wnr.0000227991.23046.b7

[17] James J Das AV Rahnenfuhrer J Ahmad I Cellular and molecular characterization of early and late retinal stem cells/progenitors: differential regulation of proliferation and context dependent role of Notch signaling. J Neurobiol 2004 61 359 76 15452852 10.1002/neu.20064

[18] Ahmad I Dooley CM Thoreson WB Rogers JA Afiat S In vitro analysis of a mammalian retinal progenitor that gives rise to neurons and glia. Brain Res 1999 831 1 10 10411978 10.1016/s0006-8993(99)01376-1

[19] Xue LP Lu J Cao Q Kaur C Ling EA Nestin expression in Muller glial cells in postnatal rat retina and its upregulation following optic nerve transection. Neuroscience 2006 143 117 27 16949759 10.1016/j.neuroscience.2006.07.044

[20] Kondo T Johnson SA Yoder MC Romand R Hashino E Sonic hedgehog and retinoic acid synergistically promote sensory fate specification from bone marrow-derived pluripotent stem cells. Proc Natl Acad Sci USA 2005 102 4789 94 15778294 10.1073/pnas.0408239102 PMC555703

[21] Das AV Bhattacharya S Zhao X Hegde G Mallya K Eudy JD Ahmad I The canonical Wnt pathway regulates retinal stem cells/progenitors in concert with Notch signaling. Dev Neurosci 2008 30 389 409 19033687 10.1159/000178017

[22] Qiu G Seiler MJ Thomas BB Wu KB Radosevich M Sadda SR Revisiting nestin expression in retinal progenitor cells in vitro and after transplantation in vivo. Exp Eye Res 2007 84 1047 59 17451684 10.1016/j.exer.2007.01.014

[23] Trimarchi JM Stadler MB Cepko CL Individual retinal progenitor cells display extensive heterogeneity of gene expression. PLoS One 2008 3 e1588 18270576 10.1371/journal.pone.0001588 PMC2220035

[24] Tomita M Mori T Maruyama K Zahir T Ward M Umezawa A Young MJ A comparison of neural differentiation and retinal transplantation with bone marrow-derived cells and retinal progenitor cells. Stem Cells 2006 24 2270 8 17008430 10.1634/stemcells.2005-0507

[25] Senatorov V Malyukova I Fariss R Wawrousek EF Swaminathan S Sharan SK Tomarev S Expression of mutated mouse myocilin induces open-angle glaucoma in transgenic mice. J Neurosci 2006 26 11903 14 17108164 10.1523/JNEUROSCI.3020-06.2006 PMC6674879

[26] Pla P Hirsch MR Le Crom S Reiprich S Harley VR Goridis C Identification of Phox2b-regulated genes by expression profiling of cranial motoneuron precursors. Neural Dev. 2008 3 14 18565209 10.1186/1749-8104-3-14 PMC2441621

[27] Pan L Deng M Xie X Gan L ISL1 and BRN3B co-regulate the differentiation of murine retinal ganglion cells. Development 2008 135 1981 90 18434421 10.1242/dev.010751 PMC2758274

[28] Klassen H Kiilgaard JF Zahir T Ziaeian B Kirov I Scherfig E Warfvinge K Young MJ Progenitor cells from the porcine neural retina express photoreceptor markers after transplantation to the subretinal space of allorecipients. Stem Cells 2007 25 1222 30 17218397 10.1634/stemcells.2006-0541

[29] Canola K Angenieux B Tekaya M Quiambao A Naash MI Munier FL Schorderet DF Arsenijevic Y Retinal stem cells transplanted into models of late stages of retinitis pigmentosa preferentially adopt a glial or a retinal ganglion cell fate. Invest Ophthalmol Vis Sci 2007 48 446 54 17197566 10.1167/iovs.06-0190 PMC2823590

[30] Aftab U Jiang C Tucker B Kim JY Klassen H Miljan E Sinden J Young M Growth kinetics and transplantation of human retinal progenitor cells. Exp Eye Res 2009 89 301 10 19524569 10.1016/j.exer.2009.03.025

[31] Akagi T Haruta M Akita J Nishida A Honda Y Takahashi M Different characteristics of rat retinal progenitor cells from different culture periods. Neurosci Lett 2003 341 213 6 12697286 10.1016/s0304-3940(03)00177-0

[32] Huang XY Yin ZQ Tan XL Characteristics of retinal stem cells from rat optic cup at embryonic day 12.5 (tailbud stage). Cell Tissue Res 2008 333 381 93 18607633 10.1007/s00441-008-0653-1

[33] Mu X Fu X Sun H Beremand PD Thomas TL Klein WH A gene network downstream of transcription factor Math5 regulates retinal progenitor cell competence and ganglion cell fate. Dev Biol 2005 280 467 81 15882586 10.1016/j.ydbio.2005.01.028

[34] Matter-Sadzinski L Puzianowska-Kuznicka M Hernandez J Ballivet M Matter JM A bHLH transcriptional network regulating the specification of retinal ganglion cells. Development 2005 132 3907 21 16079155 10.1242/dev.01960

[35] Qiu F Jiang H Xiang M A comprehensive negative regulatory program controlled by Brn3b to ensure ganglion cell specification from multipotential retinal precursors. J Neurosci 2008 28 3392 403 18367606 10.1523/JNEUROSCI.0043-08.2008 PMC2459333

[36] Oh EC Khan N Novelli E Khanna H Strettoi E Swaroop A Transformation of cone precursors to functional rod photoreceptors by bZIP transcription factor NRL. Proc Natl Acad Sci USA 2007 104 1679 84 17242361 10.1073/pnas.0605934104 PMC1780067

[37] Liu CJ Chaturvedi N Barnstable CJ Dreyer EB Retinal Thy-1 expression during development. Invest Ophthalmol Vis Sci 1996 37 1469 73 8641850

[38] Simon PD McConnell J Zurakowski D Vorwerk CK Naskar R Grosskreutz CL Dreyer EB Thy-1 is critical for normal retinal development. Brain Res Dev Brain Res 1999 117 219 23 10567740 10.1016/s0165-3806(99)00123-6

[39] Fujitani Y Fujitani S Luo H Qiu F Burlison J Long Q Kawaguchi Y Edlund H MacDonald RJ Furukawa T Fujikado T Magnuson MA Xiang M Wright CV Ptf1a determines horizontal and amacrine cell fates during mouse retinal development. Development 2006 133 4439 50 17075007 10.1242/dev.02598

